# Cortisol Co-Secretion and Clinical Usefulness of ACTH Stimulation Test in Primary Aldosteronism: A Systematic Review and Biases in Epidemiological Studies

**DOI:** 10.3389/fendo.2021.645488

**Published:** 2021-03-16

**Authors:** Kosuke Inoue, Takumi Kitamoto, Yuya Tsurutani, Jun Saito, Masao Omura, Tetsuo Nishikawa

**Affiliations:** ^1^ Department of Epidemiology, University of California, Los Angeles (UCLA) Fielding School of Public Health, Los Angeles, CA, United States; ^2^ Endocrinology and Diabetes Center, Yokohama Rosai Hospital, Yokohama, Japan; ^3^ Division of Endocrinology, Department of Medicine, Columbia University, New York, NY, United States

**Keywords:** cortisol, ACTH, primary aldosteronism, systematic review, bias, epidemiological methods

## Abstract

The hypothalamus-pituitary-adrenal (HPA) axis plays an important role in primary aldosteronism. Aldosterone biosynthesis is regulated not only by angiotensin II in the renin-angiotensin-aldosterone system, but also by adrenocorticotropic hormone (ACTH), one of the key components of the HPA axis. Although previous studies have reported cortisol cosecretion in primary aldosteronism, particularly aldosterone-producing adenoma (APA), the clinical relevance of such aldosterone and cortisol cosecretion from APA and hypertension or other metabolic disorders has not been fully established. Several somatic mutations including *KCNJ5* and *CACNA1D* are known to induce autonomous production of aldosterone in APA, and the aldosterone responsiveness to ACTH may vary according to each mutation. The ACTH stimulation test has been reported to be a useful tool to distinguish the subtypes of primary aldosteronism (e.g., unilateral vs bilateral) in some studies, but it has not been commonly applied in clinical practice due to limited evidence. Given the recent advancement of imaging, omics research, and computational approach, it is important to summarize the most updated evidence to disentangle the potential impact of cortisol excess in primary aldosteronism and whether the ACTH stimulation test needs to be considered during the diagnostic process of primary aldosteronism. In this article, we conducted a systematic review of epidemiological studies about (i) cortisol cosecretion in primary aldosteronism and (ii) the ACTH stimulation test for the diagnosis of primary aldosteronism (including subtype diagnosis). Then, we discussed potential biases (e.g., confounding bias, overadjustment, information bias, selection bias, and sampling bias) in the previous studies and introduced some advanced epidemiological/statistical methods to minimize these limitations. A better understanding of biases and epidemiological perspective on this topic would allow us to produce further robust evidence and balanced discussion about the causal mechanisms involving the HPA axis and clinical usefulness of the ACTH stimulation test among patients with primary aldosteronism.

## Introduction

Primary aldosteronism (PA) is one of the common causes of secondary hypertension, increasing the risk of cardiovascular disease (CVD) and renal events (1–4). Since the first report of a patient with adrenal adenoma concurrently producing aldosterone and cortisol (5), cortisol cosecretion in PA has received substantial attention, particularly because it may cause severe complications due to cortisol excess in addition to aldosterone excess. A recent study using a mass spectrometry-based analysis in Europe showed that cortisol cosecretion in PA was associated with adverse metabolic risk factors (6), indicating the role of glucocorticoid on an increased risk of metabolic disorders among patients with PA (7–11). Moreover, while the actual prevalence of this phenotype is not clear, some studies have suggested a relatively high prevalence of PA with hypercortisolism than expected (6, 10–15). Therefore, it is imperative to understand the current state of knowledge about cortisol cosecretion in PA, and consider whether the evaluation of cortisol excess needs be recommended in general PA screening or not.

Although the hypothalamus-pituitary-adrenal (HPA) axis plays an important role in regulating aldosterone biosynthesis, the clinical usefulness of ACTH stimulation test during the management of PA, particularly for PA subtype diagnosis (e.g., aldosterone-producing adenoma [APA] vs. bilateral aldosterone hyperplasia [BAH]), has been unclear. In general, ACTH is known to stimulate both cortisol and aldosterone secretion acutely and transiently through binding to melanocortin type 2 receptor (MC2R) (16). Previous studies have shown the high performance of classifying subtype of PA based on plasma aldosterone concentrations during ACTH stimulation test under 1-mg dexamethasone suppression test (DST) (17, 18). Other studies have also reported a larger decrease in aldosterone levels following dexamethasone suppression of HPA axis among APA than BAH (19) or among *KCNJ5*-mutated APAs than *KCNJ5* wild-type APAs (20), indicating the regulating role of endogenous ACTH on aldosterone secretion among patients with PA. Because lateralization of hyperaldosteronism is crucial to determine the target treatment (i.e., adrenalectomy or medication therapy) (21), there has been a great interest in the clinical benefit of conducting ACTH stimulation test during the PA confirmation process before AVS—a gold standard but more invasive and challenging procedure for PA subtype diagnosis.

Over the last several decades, observational studies have played a key role in PA research. Because exposure or treatment is not randomized in observational studies, we always need to be careful about confounding bias, a bias due to unmeasured common causes of exposure and outcomes (22). This is also the case in the “big data” analysis or machine learning-based approach because they do not guarantee the adjustment of a sufficient set of confounders and could rather introduce *precisely wrong* conclusion due to small variance of the estimates under the presence of bias. Moreover, both observational studies and randomized controlled trials suffer from other sources of bias including information bias (e.g., measurement error of hormone levels, self-report of history of hypertension and medications) (23) and selection bias (e.g., loss to follow-up, selection of study sample based on both exposure and outcome assessment) (24). Limited generalizability due to sampling bias is another issue of previous PA-related studies because most of the studies have been conducted at single-center and/or specific cohorts.

This review has two goals. First, we will provide updated summaries of epidemiological studies about cortisol cosecretion in primary aldosteronism and the clinical usefulness of the ACTH stimulation test to diagnose primary aldosteronism and/or its subtypes. Then, we will explain possible biases (e.g., confounding bias, overadjustment, information bias, selection bias, and sampling bias) in these epidemiological studies and some methods to minimize such limitations in future PA-related studies. Recognizing these biases and applying the advanced epidemiological methods would help us to build a further balanced and profound discussion on this topic—cortisol cosecretion and clinical usefulness of the ACTH stimulation test in PA.

## Cortisol Cosecretion in Primary Aldosteronism

**Figure 1:**
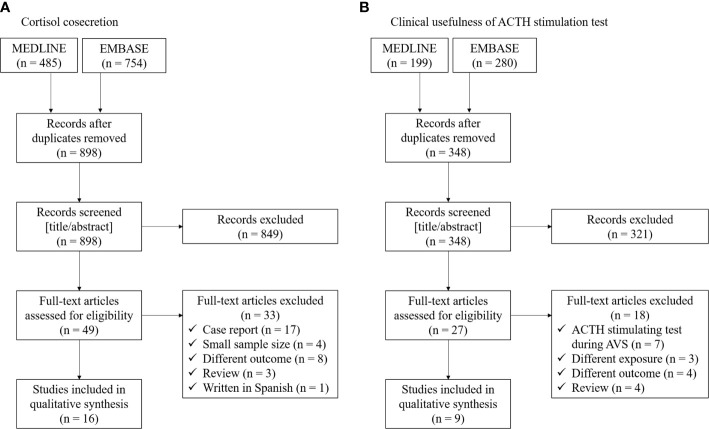
Flow of studies through review for **(A)** cortisol cosecretion and **(B)** clinical usefulness of ACTH stimulation test in primary aldosteronism.

Recently, there is increasing literature regarding cortisol cosecretion in PA and its impact on CVD risk factors (6, 25, 26). We conducted literature searches between January 2000 and November 2020 using the electronic databases MEDLINE and EMBASE for cohort studies investigating the cortisol cosecretion in primary aldosteronism. The following search terms were applied: (“cortisol” OR “cortisolemia” OR “Cushing”) AND (“primary aldosteronism” OR “aldosterone-producing adenoma”). We extracted the following information: first author name, publication year, region of study, populations, exposures/comparators, outcomes, and study design. We restricted studies to those with a sample size ≥10 (to avoid case reports) and written in English. Flow of studies through review and summary of the identified 16 studies are shown in **Figure 1A** and **Table 1** (6, 10–15, 27–35).

**Table 1:** Summary of epidemiological studies about cortisol cosecretion in primary aldosteronism (PA).

Author	Year	Region	Populations	Exposures/Comparators	Characteristics/Outcomes	Study design
Hiraishi et al. (12)	2011	Japan	38 patients with PA	Coexistence of PA and subclinical Cushing’s syndrome^a^ (n=8)	Clinical and histopathological characteristics	Cross-sectional study
Nakajima et al. (27)	2011	Japan	76 patients with PA	Coexistence of PA and subclinical cortisol hypersecretion (n=22, serum cortisol levels during 1 mg DST ≥3.0 μg/dl)	Clinical characteristics including a history of cardiovascular events	Cross-sectional study
Fallo et al. (28)	2011	Italy	76 patients with PA	Coexistence of PA and subclinical cortisol hypersecretion (n=3, serum cortisol levels during 1 mg DST >1.8 μg/dl)	Clinical and histopathological characteristics	Cohort study
Fujimoto et al. (13)	2013	Japan	39 PA patients	Coexistence of PA and subclinical Cushing’s syndrome^a^ (n=5)	Clinical and histopathological characteristics	Cross-sectional study
Arlt et al. (6)	2017	Germany	174 patients with PA^b,c^	Cortisol cosecretion (24-h cortisol and total glucocorticoid outputs collected by quantitative gas chromatography-mass spectrometry)	Metabolic risk factors (BMI, blood pressure, fasting plasma glucose and insulin, 2-h glucose values during 75-g oral glucose tolerance test, HbA1c, total cholesterol, HDL, and triglycerides)	Cohort study
Inoue et al. (29)	2017	Japan	30 patients with APA and serum cortisol levels during 1 mg DST <3.0 μg/dl	Adrenalectomy	The change in serum cortisol levels during the 1-mg DST before and after adrenalectomy and histological characteristics	Cohort study
Tang et al. (14)	2018	China	414 patients with APA	Coexistence of APA and subclinical cortisol hypersecretion (n=22, serum cortisol levels during 1 mg DST >1.8 μg/dl)	Clinical and histopathological characteristics	Cross-sectional study
Adolf et al. (30)	2019	Germany	73 patients with PA ^c^	Cortisol cosecretion (24-h total glucocorticoid outputs collected by quantitative gas chromatography-mass spectrometry)	Left ventricular hypertrophy	Cohort study
Ohno et al. (31)	2019	Japan	527 patients with bilateral PA. ^d^	Bilateral PA cases with adrenal tumors (n=196) and without adrenal tumors (n=331)	Hormone levels including serum cortisol levels during 1 mg DST and clinical complications	Cross-sectional study
Kometani et al. (32)	2019	Japan	16 APAs	Coexistence of APA and subclinical cortisol hypersecretion (n=6, serum cortisol levels during 1 mg DST and at midnight >1.8 μg/dl)	Genetic and epigenetic characteristics	Cross-sectional study
Bhatt et al. (33)	2019	UK	25 patients with PA	Coexistence of PA and subclinical cortisol hypersecretion (n=4, serum cortisol levels during 1 mg DST >1.8 μg/dl)	Metabolic risk factors (ALT, total cholesterol, HDL, LDL and mean arterial blood pressure)	Cross-sectional study
Gerards et al. (10)	2019	Germany	161 patients with PA^c^	Coexistence of PA and subclinical cortisol hypersecretion (n=125, serum cortisol levels during 1 mg DST >1.8 μg/dl, or late-night salivary cortisol >1.45 ng/ml, or 24-h urinary free cortisol >150 μg/24h)	Glucose homeostasis evaluated by the standard oral glucose tolerance test	Cohort study
Akehi et al. (11)	2019	Japan	890 patients with PA who conducted 1 mg DST ^d^	Coexistence of PA and subclinical cortisol hypersecretion (n=209, serum cortisol levels during 1 mg DST >1.8 μg/dl)	Prevalence of diabetes	Cross-sectional study
Handgriff et al. (34)	2020	Germany	97 patients with PA^c^	Coexistence of PA and subclinical cortisol hypersecretion (n=72, serum cortisol levels during 1 mg DST >1.8 μg/dl, or late-night salivary cortisol >1.5 ng/ml, or 24-h urinary free cortisol >150 μg/l [before 2015] or >83 μg/l [after 2015])	The kinetics of anti-thyroid peroxidase and thyroglobulin antibody before and after the therapy initiation	Cohort study
O’Toole et al. (35)	2020	UK	144 patients with PA	Coexistence of PA and subclinical cortisol hypersecretion (n=21, serum cortisol levels during 1 mg DST >1.8 μg/dl)	Parameters and interpretation in adrenal venous sampling	Cross-sectional study
Peng et al. (15)	2020	Taiwan	82 patients with APA	Coexistence of APA and subclinical cortisol hypersecretion (n=22, serum cortisol levels during 1 mg DST >1.8 μg/dl)	Clinical and biochemical outcomes after adrenalectomy	Cohort study

BMI, body mass index; HbA1c, glycohemoglobin; HDL, high-density lipoprotein cholesterol; APA, aldosterone-producing adenoma; A/CPA, aldosterone and cortisol-coproducing adenoma; DST, dexamethasone suppression test.

a Subclinical Cushing’s syndrome was defined based on diagnostic criteria proposed by the Research Committee for Adrenal Diseases supported by the Japanese Ministry of Health, Labor and Welfare: the presence of adrenal incidentaloma, lack of Cushingoid features, and normal basal but autonomous cortisol secretion with no suppression of cortisol by low-dose (1 mg) and high-dose (8 mg) DST (>3 μg/dL and >1 μg/dL, respectively), and at least one of the following additional endocrine data: 1) suppressed plasma ACTH (<10 pg/mL) and/or decreased response of ACTH after CRH stimulation, 2) loss of cortisol diurnal rhythm, 3) decreased serum DHEA-S levels, 4) unilateral uptake of ^131^I-adosterol by adrenal scintigraphy.

b This study also included 162 healthy controls, 56 patients with endocrine inactive adrenal adenoma, 104 patients with mild subclinical, and 47 with clinically overt adrenal cortisol excess as comparison groups.

c The German Conn’s registry.

d The Japan Primary Aldosteronism Study.

Previous studies have shown a relatively high prevalence of subclinical Cushing’s syndrome or subclinical hypercortisolemia among patients with PA although the definition of hypercortisolemia differs across studies and countries (**Table 1**). In 2009, Piaditis et al. reported 12.1% of patients with adrenal incidentalomas had PA with hypercortisolemia (36). Since then, several studies from the Asian cohort have reported a 5%–27% prevalence of subclinical hypercortisolemia among patients with PA or APA (11–15). A recent multicenter cohort study in Germany showed even 77.6% of subclinical hypercortisolemia among 161 patients with PA (10). While the difference in clinical characteristics between PA patients with and without hypercortisolemia is still not clear (mainly due to the small sample size in each previous study) and may vary by geographical area and definitions, most of these studies showed larger tumor size among PA patients with subclinical hypercortisolemia than those without.

In 2014, monoclonal antibodies against human CYP11B1 and CYP11B2 were developed and their expression was reported in APAs, indicating the cortisol synthesis in such adrenal tumors (37, 38). This finding was supported by a small cohort study reporting a significant decrease in serum cortisol levels during 1 mg DST after the resection of APAs (29) and a large mass spectrometry-based analysis showing higher excretion of cortisol and total glucocorticoid metabolites in the PA group compared with the healthy control group (6). Moreover, a previous case series showed that the majority of APAs with subclinical hypercortisolemia was composed mainly of ZF-like cells (39). Given that ZF comprise larger cells that synthesize glucocorticoids compared with ZG that synthesize aldosterone (40), such histopathological feature may be related to the large tumor size in APAs with subclinical hypercortisolemia, that requires further investigation.

Recent studies also have reported health outcomes related to autonomous cortisol secretion in APAs. The above-mentioned mass spectrometry-based analysis also found the correlation between glucocorticoid output and several metabolic markers including body mass index, waist circumference, insulin resistance, high-density lipoprotein, and diastolic blood pressure (6). Tang et al. showed higher proportions of cardiovascular complications, glucose intolerance/diabetes, and osteopenia/osteoporosis among patients with APAs and cortisol producing adenoma (CPA) than patients with pure APAs (14). Adolf et al. scrutinized the possible impact of cortisol excess on cardiac remodeling among PA patients (30). Across patients in the German Conn’s registry, they found the association of total glucocorticoid excretion with the decrease in LVMI a year after the initiation of PA treatment (30). The association between subclinical hypercortisolemia and impaired glucose metabolism in patients with PA was also reported by other cohort studies (10, 11). These results support the adverse effect of glucocorticoid excess on health through the activation of not only glucocorticoid receptor but also mineralocorticoid receptor (MR) through the impaired conversion of cortisol to its MR-inactive cortisone (41). Another study using the same German registry showed significant increases in anti-TPO levels after adrenalectomy in unilateral PA patients with cortisol cosecretion but the trend was not found in unilateral PA patients without cortisol cosecretion and bilateral PA patients receiving mineralocorticoid antagonist therapy (34). Their findings generate the hypothesis that cortisol cosecretion among unilateral PA may exhibit the immunosuppressive effect. Given these findings, it would be important to evaluate the cortisol cosecretion among PA patients in routine clinical practice. To produce robust evidence, more research is warranted to clarify whether the adverse health outcomes of APAs with subclinical hypercortisolemia differ between those with and without CPA.

The possible influence of mild cortisol excess in PA on the interpretation of AVS has been suggested in prior literature. In general, cannulation success is evaluated by selectivity index (the ratio of cortisol concentration for each adrenal vein and inferior vena cava [IVC]), and the lateralization is evaluated by lateralization index (the aldosterone to cortisol ratio on the dominant side with excess aldosterone secretion over aldosterone to cortisol ratio on the non-dominant side) and contralateral suppression index (the aldosterone to cortisol ratio on the non-dominant side over aldosterone to cortisol ratio in IVC) (21). Based on the findings from AVS in patients with CPA, but without PA, Goupil et al, suggested that autonomous unilateral cortisol hypersecretion could confound the accuracy of AVS by affecting the aldosterone/cortisol ratio in the adrenal vein (i.e., selectivity index, lateralization index, and contralateral suppression index), leading to misdiagnosis and suboptimal management (42). However, a recent single-center study focusing on PA with mildly elevated cortisol levels (mean serum cortisol levels during the 1-mg DST = 3.3 μg/dl) showed that the rates of cannulation success or lateralization did not differ between PA patients with mild cortisol excess and those without cortisol excess during AVS with ACTH stimulation (blood sample was obtained 1 h after the stimulation) (35). A multi-center cohort study on this topic would help us to validate their findings and further understand which AVS parameters are useful for the management of PA with cortisol cosecretion.

Lastly, some studies have investigated the genetic mutation of APA with cortisol cosecretion. A recent study based on a Taiwanese database showed that the presence of *KCNJ5* mutations was significantly lower among APA patients with subclinical hypercortisolemia than those without (15). Their immunohistochemistry analysis revealed the lower CYP11B1 expression among *KCNJ5* mutated APAs, which generally display a ZF-like phenotype (43). Although the underlying mechanism is unclear, enhanced 18-oxocortisol synthesis in *KCNJ5* mutated APAs may contribute to the findings of the lower cortisol levels in the mutated APAs than *KCNJ5* wild-type APAs. They also found that patients with subclinical hypercortisolemia and *KCNJ5* wild-type APAs exhibited the lowest complete clinical success rate (36.8%) after adrenalectomy (43), highlighting the importance of detecting these phenotypes for the management of APAs (15). Genetic and epigenetic analysis in a Japanese single-center study showed that APA with hypercortisolemia was not associated with the prevalence of *KCNJ5* mutations but associated with lower DNA methylation rate of the CYP11B1 promoter than APA without hypercortisolemia (32). However, this study was based on only 16 APAs and the causal relationship between DNA methylation and the regulation of gene expression is still unclear. Given that some somatic mutations, such as *CTNNB1* and *PRKACA* (44, 45), have also been reported in both APAs and CPAs, future research about these mutations and cortisol cosecretion among PA patients would be warranted.

## ACTH Stimulation Test for the Diagnosis of Primary Aldosteronism

Aldosterone production from the adrenal glomerulosa is regulated by ACTH as well as potassium, angiotensin II, and serotonin (46). Previous biological studies showed that acute ACTH administration increases plasma aldosterone through binding to MC2R (47), suggesting that ACTH may play a role in inappropriate hypersecretion of aldosterone in some PAs (16). A previous study showed aldosterone responsiveness to even very low dose ACTH (i.e., 0.003 IU), followed by a treadmill test (48). Since the first report of the ACTH stimulation test for patients with PA in 1978 (5), several epidemiological studies have investigated whether the ACTH stimulation test is useful to diagnose PA or its subtype. We conducted literature searches between January 2000 and November 2020 using the electronic databases MEDLINE and EMBASE for cohort studies investigating the cortisol cosecretion in primary aldosteronism. The following search terms were applied: (“ACTH” OR “adrenocorticotropic hormone” OR “corticotropin”) AND (“primary aldosteronism” OR “aldosterone-producing adenoma”). We extracted the following information: first author name, publication year, region of study, populations, exposures/comparators, outcomes, and study design. We restricted studies to those with a sample size ≥10 (to avoid case reports) and written in English. Flow of studies through review and summary of the identified 9 studies are shown in **Figure 1B** and **Table 2** (17–19, 49–54).

**Table 2:** Summary of epidemiological studies about ACTH stimulation test for the definite and the subtype diagnosis of primary aldosteronism (PA).

Author	Year	Region	Populations	Exposures/Comparators	Outcomes	Study design
Sonoyama et al. (17)	2011	Japan	39 patients with PA (unilateral, n=23; bilateral, n=16) and 20 patients without PA	ACTH stimulation (0.25mg [25IU] of cosyntropin) with 1 mg DST	The predictive accuracy for the subtype diagnosis of PA (i.e., unilateral or bilateral).	Cohort study
Jiang et al. (18)	2015	China	95 patients with PA (unilateral, n=56; bilateral, n=39)	ACTH stimulation (50IU) with 1 mg DST	The predictive accuracy for the subtype diagnosis of PA	Cohort study
Umakoshi et al. (49)	2016	Japan	121 patients with PA (unilateral, n=34; bilateral, n=87) and 66 patients without PA	ACTH stimulation (25IU)	The predictive accuracy for the definite and subtype diagnosis of PA	Cohort study
Terui et al. (50)	2016	Japan	138 patients with PA (unilateral, n=41; bilateral, n=57; no AVS, n=40) and 19 patients without PA	ACTH stimulation (25IU)	The predictive accuracy for the definite and subtype diagnosis of PA	Cohort study
Inoue et al. (51)	2017	Japan	30 patients with PA (unilateral, n=13; bilateral, n=17) and 18 patients without PA	ACTH stimulation (25IU) with and without 1 mg DST	The predictive accuracy for the definite and subtype diagnosis of PA	Cohort study
Moriya et al. (52)	2017	Japan	76 patients with PA (unilateral, n=17; bilateral, n=59)	ACTH stimulation (25IU)	The predictive accuracy for the subtype diagnosis of PA	Cohort study
Kita et al. (53)	2018	Japan	40 patients with PA (unilateral, n=22; bilateral, n=18)	ACTH stimulation (25IU)	The predictive accuracy for the subtype diagnosis of PA	Cohort study
Kidoguchi et al. (54)	2020	Japan	123 (unilateral, n=27; bilateral, n=96)	ACTH stimulation (25 IU)	The predictive accuracy for the subtype diagnosis of PA	Cohort study
St-Jean et al. (19)	2020	Canada	43 (unilateral, n=28; bilateral, n=11; undefined, n=4)	ACTH stimulation (25 IU)^a^	Aldosterone responsiveness after the stimulation	Cohort study

DST, dexamethasone suppression test.

a The effect of endogenous ACTH on aldosterone secretion was indirectly evaluated by the relative percent of suppression of aldosterone following dexamethasone suppression during at least 48 h.

In 1995, Stowasser et al. reported higher plasma aldosterone response after ACTH stimulation under dexamethasone suppression among angiotensin II responsive APA (n=16) or angiotensin II unresponsive APA (n=11) compared with BAH (n=19) (55). Although several studies mostly from Japan have shown moderate to high predictive performance (area under the curve [AUC] ranges from 0.70 to 0.95) to differentiate APA from BAH by using ACTH stimulation test (17, 18, 49–52, 54) over the last two decades, these findings are not comparable across studies due to the lack of uniformity in protocols in each study (e.g., ACTH dosage, with or without 1 mg DST, different cut-off time or values, etc), inconsistent definition of outcomes (e.g., diagnosis of APA and BAH based on AVS, histopathological findings, etc.), and study design (sample size, single-center or multi-center, prospective or retrospective, etc.). These limitations have made the clinical usefulness of the ACTH stimulation test inconclusive. For example, we previously demonstrated that the predictive performance of the ACTH stimulation test for subtype diagnosis of PA could vary by the definition of lateralization during AVS with ACTH stimulation (51); i.e., whether evaluating the ratio of aldosterone to cortisol at adrenal central veins (21) or evaluating the absolute value of aldosterone at adrenal tributary veins (56). Compared with the subtype diagnosis of PA, the evidence about the definite diagnosis of PA has been very limited and has not been consistent (49–51). As the current literature on this topic is mostly based on small sample size and a single-center or a specific country, further investigations with larger sample size from multi-center cohort are needed to understand whether the ACTH stimulation test should be routinely included during the process of PA diagnosis and which parameters are useful to minimize the use of AVS which is more invasive and challenging than peripheral blood examination.

Whether ACTH stimulation is recommended during AVS for subtype diagnosis or not is another important topic in PA management. ACTH stimulation may maximize cortisol secretion from the adrenal gland and minimize the pulsatile adrenocortical hormone secretion. A recent meta-analysis showed that AVS with ACTH stimulation did not significantly reduce the number of incorrect lateralization but significantly reduced the number of unsuccessful cannulations compared with AVS without ACTH stimulation (57). However, the evidence is still limited and under debate which is beyond the scope of this review and can be found in prior review literature (58).

Aldosterone responsiveness to ACTH may vary by a genetic mutation. Our group recently reported a larger decrease in aldosterone levels during 1 mg DST among *KCNJ5* mutated APAs than *KCNJ5* wild-type APAs, indicating that the ACTH pathway may be more sensitive and activated among *KCNJ5* mutated APAs (20). We also reported that aldosterone levels were more responsive to ACTH stimulation among APAs with somatic mutation of *CACNA1D* than those without the mutation (59). APAs with these mutations are likely to consist of ZF-like clear cells than others, and ZF cells are responsible for cortisol secretion and express a higher level of ACTH receptor than ZG cells (59, 60). Given that nearly 90% of APAs had somatic mutations based on the CYP11B2 immunohistochemistry-guided, full gene-based next-generation sequencing (61–63), future multi-center studies are warranted to identify the heterogeneous aldosterone response to synthetic ACTH by these genetic mutations.

## Biases in Epidemiological Studies Related to Primary Aldosteronism

Although these previous studies were well-conducted and prismatic, they have suffered from biases that are sometimes substantial. Herein, we describe the common types of biases (confounding, overadjustment, information bias, selection bias, and sampling bias) in epidemiological studies related to PA by using directed acyclic graphs (DAGs), a graphical tool to represent causal relationships between variables by linking them through single-headed arrows (64). In this section, to make a simple explanation, we assume that the exposure of interest is PA with subclinical hypercortisolemia (PA/SH) and the outcome of interest is CVD.

### Confounding

**Figure 2:**
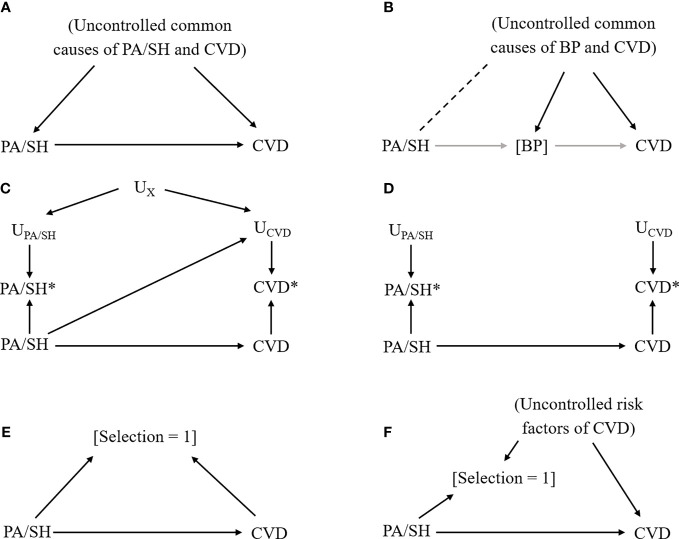
Causal diagram representing each bias scenario in primary aldosteronism study. Notation: Exposure, primary aldosteronism/subclinical hypercortisolemia (PA/SH); Outcome, cardiovascular disease (CVD); solid arrow from X to Y, the causal effect of X on Y; [X], conditioning on X; dash line between X and Y, the nonexistent association between X and Y that could be introduced by conditioning on a variable affected by X and Y; U_X_, factors that affect the measurement of X; X*, measured X (which could be different from the actual X). **(A)** Confounding bias due to uncontrolled common causes of exposure (PA/SH) and outcome (CVD). **(B)** Overadjustment and collider bias due to conditioning on an intermediate variable (blood pressure [BP]) that underestimate the total effect of PA/SH on CVD and introduce additional bias by linking PA/SH and uncontrolled common causes of BP and CVD (dashed line). **(C)** Measurement error, dependent (i.e., common factors affect measurements of exposure and outcome) and differential (i.e., exposure affects measurements of outcome, or vice versa). **(D)** Measurement error, independent and nondifferential. **(E)** Selection bias due to selecting participants by exposure and outcome status. **(F)** Selection bias due to loss to follow-up.

In observational studies, we generally cannot rule out the possibility of unmeasured confounding. This is also the case for PA studies when some common causes of PA/SH and CVD are missing (**Figure 2A**). To minimize this bias, researchers should include variables that are causes of the exposure and related to the outcome, or variables that are causes of the outcome and related to the exposure (65). If some important variables are missing, quantitative bias analysis may need to be considered to investigate the potential impact of uncontrolled confounding by the unmeasured confounders (22). This method can also be applied in the meta-analysis of observational studies (66, 67). Calculating E-value is an alternative approach to simply assess how much confounding is needed to explain away the observed association (68). Furthermore, if there is information on a genetic mutation that would affect CVD only through PA/SH phenotype and does not modify the effect of PA/SH on CVD, mendelian randomization may be considered to obtain a less biased estimate (69).

### Overadjustment and Collider Bias

Another important point to select covariates in the model is overadjustment. Controlling for intermediate variable such as blood pressure (BP) that lies within the causal pathway between PA/SH and CVD would introduce a biased estimate (**Figure 2B**) because we (i) fail to include the effect of PA/SH on CVD that is mediated through BP, and (ii) introduce the nonexistent relationship between PA/SH and CVD through uncontrolled BP-CVD confounders by controlling for BP, so-called “collider bias” (70, 71). A data-driven approach (including machine learning algorithms) is sometimes useful to efficiently select covariates included in the model, but does not take account of this bias, and therefore, researchers need to carefully select the potential confounders based on prior knowledge and causal diagram (65).

When there is an interest to estimate the mediation effect of such intermediate variables for the PA/SH-CVD association, causal mediation analysis may be a powerful tool to answer such research questions. For example, using causal mediation analysis, a recent study showed that elevated BP mediated 45% of the total association between serum aldosterone levels and coronary artery calcium score among general adults from a multi-ethnic group (72). This finding provided novel insight into both the direct effect (not through BP) and indirect effect (through BP) of elevated aldosterone on subclinical atherosclerosis. Meanwhile, it is also noteworthy that causal mediation analysis requires several strong assumptions and limitations. One of the hurdles of applying this method in PA studies is that relatively large sample size is generally needed to find the significant direct and indirect effect of the exposure on the outcome. Further details on this method can be found in the tutorial and methodological review paper (73).

Related to the overadjustment issue, temporal ordering between exposure, confounders, and outcome is crucial to obtain a less biased estimate. In PA studies, given the negative feedback system of RAAS and the HPA axis, it is often challenging to assume which variables (e.g., biomarker concentrations and medical conditions) occurred first at cross-sectional data. When the variables can be both confounders and mediators, researchers need to carefully interpret the results of stratified analysis by such variables because the seemingly heterogeneous findings could be biased (74). Temporal ordering can be established in longitudinal cohorts where the extraction of biosample and other information precedes the outcome assessment.

### Measurement Error (Information Bias)

Because analyses are generally based on data, we need to be careful about the measurement error or misclassification of variables (23). When exposure status (i.e., whether subjects have PA/SH or not) affects the reporting of CVD and/or there are common features between exposure assessment and outcome assessment (e.g., physician diagnosis, subjects’ awareness of diseases, subjects’ cognitive performance, etc), our estimate would be biased (**Figure 2C**). For example, this type of bias could easily happen when the exposure assessment involves the results of the ACTH stimulation test in peripheral blood and the outcome assessment involves the results of ACTH stimulation during AVS. Recall bias is a similar type of bias that occurs when PA/SH assessment is affected by the presence of CVD. If the error for measured PA/SH status (or CVD status) is independent of the true CVD status (or PA/SH status) and there are no common features that affect PA/SH and CVD measurement (**Figure 2D**), the bias is generally considered to be towards the null (i.e., underestimate the effect) particularly when the exposure is binary.

### Selection Bias and Loss to Follow-Up

If the study restricts patients to those with confirmatory tests for PA/SH and the selection is also affected by health outcome status or its risk factors (which is often the case in a case-control study), such selection introduces collider bias limiting internal validity (**Figure 2E**). Another example is a loss to follow-up in a cohort study or a randomized controlled trial when enrollees drop the study due to health conditions related to CVD, as known as attrition bias (**Figure 2F**). Multiple imputations for missing data or inverse probability weighting approach would be helpful to minimize this type of bias when missing mechanisms can be explained by measured covariates (75).

### Sampling Bias

Sampling bias is also caused by selecting participants that limit the generalizability of the study findings. Sampling bias needs to be considered in almost all studies (including clinical trials) because the study sample is often different from the population to which clinical interventions or guidelines are targeted in the real-world. This bias requires attention in PA studies given the different prevalence of PA/SH and somatic mutations in APA across countries (62, 63, 76); i.e., study results from a specific region may not simply be applied to other regions. Moreover, as shown in our literature review, some topic has tended to be heavily interested in a specific region or country, that also limits the external validity. Recently, the concept and method of generalizability and transportability have received renewed interest in causal inference literature to extend the study findings to the target population (77, 78).

## Conclusion

In this review paper, we summarized the current state of knowledge about primary aldosteronism with cortisol cosecretion and the role of ACTH stimulation test in PA diagnosis. In summary, there is increasing evidence about the relatively high prevalence of cortisol cosecretion in PA and its potential influence on adverse health outcomes. Further studies are warranted to clarify whether there is a clinically useful cut-off points for cortisol cosecretion (i.e., serum cortisol levels during the 1-mg DST) for the PA management. The clinical usefulness of the ACTH stimulation test for PA (subtype) diagnosis has been suggested by some studies, but the evidence is limited to make a specific recommendation in clinical practice. Multicenter studies using the uniform protocol and consistent definition are needed to produce robust evidence on this topic. Moreover, heterogeneous response to ACTH signaling among patients with PA according to genetic mutations should be further investigated. We also explained the common type of biases in these epidemiological studies and also introduced some advanced methods to minimize the biases. As these epidemiological perspectives have not sufficiently been considered in PA-related literature, we hope this review would contribute to a better understanding of possible biases in epidemiological studies and would help clinicians and researchers to produce further robust epidemiological evidence and balanced discussion on these topics.

## Data Availability Statement

The original contributions presented in the study are included in the article/supplementary material. Further inquiries can be directed to the corresponding author.

## Author Contributions

Conceptualization, KI, TK, and TN. Methodology, KI. Formal analysis, KI. Investigation, KI, TK, and TN. Writing—original draft preparation, KI. Writing—review and editing, KI, TK, YT, JS, MO, and TN. Visualization, KI. Supervision, TN. All authors contributed to the article and approved the submitted version.

## Funding

KI was supported by the National Institutes of Health (NIH)/NIDDK grant F99DK126119, Toffler award at UCLA, and the Honjo International Foundation Scholarship. This article does not necessarily represent the views and policies of the NIH. The funders had no role in the design and conduct of the study, collection, management, analysis, and interpretation of the data, preparation, review, or approval of the manuscript, and decision to submit the manuscript for publication.

## Conflict of Interest

The authors declare that the research was conducted in the absence of any commercial or financial relationships that could be construed as a potential conflict of interest.
